# Mechanically Regulated Secretion: How Physical Forces Instruct the Secretory Pathway and Remodel the Secretome

**DOI:** 10.1002/advs.78102

**Published:** 2026-10-06

**Authors:** Domenico Russo, Seetharaman Parashuraman, Maria Luigia Maresca, Angela Francesca Dinoi, Alessia Calcagnì, Riccardo Rizzo

**Affiliations:** ^1^ Institute of Endotypes in Oncology, Metabolism and Immunology “G. Salvatore” (IEOMI) National Research Council Naples Italy; ^2^ Institute of Nanotechnology National Research Council (CNR‐NANOTEC) Lecce Italy; ^3^ Tecnomed Puglia Tecnopolo per la medicina di precisione (Biotech Lecce Hub) Lecce Italy; ^4^ Telethon Institute of Genetics and Medicine (TIGEM) Pozzuoli Italy; ^5^ Department of Translational Medical Sciences Federico II University Naples Italy

**Keywords:** disease, ECM, fibrosis, mechanical cues, mechanosensing, mechanotransduction, secretory pathway, trafficking

## Abstract

Cells do not secrete in a vacuum: they continuously interpret mechanical and chemical stimuli. This “cell sociology” drives collective behaviors and communication networks, allowing cells to process information from their surroundings. Over the past decade, growing evidence shows that all major secretory organelles are responsive to mechanical cues, overturning the view of secretion as a purely biochemical process. Cues like extracellular matrix (ECM) stiffness, cell shape, membrane tension, and tissue deformation dynamically tune trafficking at every step, remodeling endoplasmic reticulum (ER) exit sites, reshaping Golgi architecture, redirecting sorting at the trans‐Golgi network (TGN), repositioning endolysosomes, and controlling exosome release. This mechanical control operates through complementary transcriptional and post‐translational mechanisms, including GTPase activation, kinase cascades, cytoskeletal tension, and lipid remodeling, enabling rapid organelle reconfiguration. A mechano‐secretory feedback loop exists: the physical microenvironment reorganizes the secretory pathway, and the resulting secretome changes reshape ECM composition and tissue mechanics. While supporting physiological homeostasis, this loop dysregulation drives fibrosis, cancer progression, and immune dysfunction. In this review, we integrate recent major findings to provide a coherent framework for understanding how mechanical forces reshape the secretory pathway and influence tissue function and disease.

## Graphical abstract

Mechanical cues, including ECM stiffness, stretch, compression, shear stress, and traction forces, remodel trafficking across the secretory pathway, from the ER and Golgi to endolysosomal compartments and the plasma membrane. The resulting secretome reshapes ECM composition and tissue mechanics, establishing feedback loops that support homeostasis or, when dysregulated, drive fibrosis, cancer, and immune dysfunction.

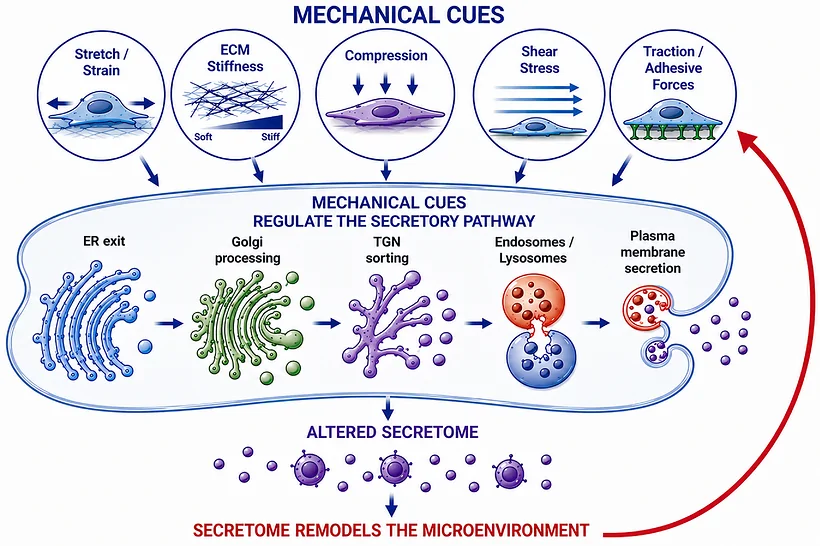

## 1 Introduction: The Mechanical Dimension of Secretion

Cells operate as members of highly coordinated communities, continuously exchanging information with their microenvironment. These social relations [1], together with the networks that sustain tissue function, rely on the secretory machinery as a bidirectional platform for intercellular signaling and intracellular logistics [2, 3, 4]. How microenvironmental mechanics tune this network is an emerging frontier in cell biology, with mechanical cues now recognized as key regulators of cellular behavior alongside transcriptional programs [5, 6].

Differences in tissue mechanics often define the trajectory of aging and disease progression [7, 8]. Pathological changes in ECM organization and rigidity may perturb mechanochemical signaling and alter secretory outputs, potentially affecting communication with surrounding cells. We hypothesize that, when this information flow becomes dysregulated, maladaptive secretory programs may contribute to the progression of fibrosis, cancer, and age‐associated disorders. Secretion and tissue mechanics are linked by a mechanochemical feedback loop: secretion remodels ECM composition, while ECM mechanics reciprocally regulate secretory function [9, 10]. Mechanical cues thereby act as collective “social signals” that coordinate tissue‐wide responses: rigid environments promote secretory programs that reinforce stiffness, whereas compliant matrices favor plasticity and remodeling [9]. When dysregulated, this feedback loop becomes pathological [11, 12, 13], also affecting the magnitude and the spatial organization of secretion, including the establishment of epithelial polarity [14].

Intracellular trafficking has traditionally been viewed as a sequence of biochemically defined steps, ER export, Golgi processing, TGN sorting, and membrane fusion [15], independent of mechanical inputs. Yet cells are continuously subjected to mechanical stimuli such as stretch, compression, shear, and substrate stiffness [16], and perturbations in tissue mechanics or mechanosensing often precede pathological transformations in cancer, fibrosis, and cardiovascular disease [7, 8]. Similarly, mechanobiology has traditionally focused on force sensing at the plasma membrane, cytoskeleton, and nucleus [17]. While integrins, focal adhesions, and Yes‐associated protein (YAP)/Transcriptional coactivator with PDZ‐binding motif (TAZ) signaling constitute the canonical mechanotransduction routes [11, 18], recent work has highlighted the endomembrane system as a crucial mechanoresponsive compartment [17, 19, 20]. The ER and Golgi dynamically remodel in response to mechanical inputs [9, 21], and mechanical stimuli further regulate membrane tension, vesicle budding and fusion, and cytoskeletal coupling to modulate protein flow through these pathways [22].

Mechanosensitive responses unfold across multiple timescales. Rapid physical challenges, such as immune cell extravasation or acute tissue injury, trigger immediate, force‐dependent modifications in vesicle trafficking, which are subsequently consolidated by long‐term transcriptional reprogramming [23]. These adaptations underscore the inherently social nature of tissue biology: cells continuously adjust their secretory behaviour to maintain coherent responses to physical challenges: for instance, stretched tissues stimulate rapid secretion of ECM proteins and growth factors, whereas metastatic cancer cells navigating confined spaces tune their secretome to remodel barriers and instruct stromal cells [24]. Because ECM stiffness reflects a dynamic balance between synthesis, crosslinking, and degradation [25, 26], pathological ECM remodeling (e.g., tumor desmoplasia or tissue fibrosis) drives progressive stiffening [27]. This establishes a vicious cycle in which increased rigidity enhances secretion of ECM components and crosslinking enzymes, further amplifying stiffness and disrupting communication [27, 28].

Mechanotransduction from the ECM to intracellular compartments proceeds through integrin‐mediated adhesion complexes linked to the actin cytoskeleton [29, 30], activating small GTPases and YAP/TAZ‐dependent transcription [11, 31]. These signals propagate inward to the endomembrane system, where changes in membrane tension link ECM stiffness to secretory dynamics [32].

This review focuses on how mechanical cues are sensed and integrated by the core organelles of the secretory pathway. Throughout this manuscript, we distinguish among physically distinct inputs, including substrate stiffness, cell spreading, stretch, membrane tension, compression, shear stress, and direct force on organelle membranes, which engage non‐interchangeable transmission routes. Furthermore, we categorize findings across three levels of evidence: direct organelle mechanosensing, indirect mechanotransduction via upstream signaling, and correlative associations, where physical changes accompany secretory phenotypes without a fully defined molecular mechanism (Table 1). Conceptually, we frame secretion and tissue mechanics as reciprocally coupled: mechanical inputs reshape the secretory machinery, and the resulting secretory outputs remodel the mechanical microenvironment in turn, a feedback loop central to both homeostasis and disease. In the sections that follow, we examine how mechanical forces reshape ER exit, Golgi remodeling, cargo sorting, membrane fusion, and extracellular vesicle (EVs) release, and how these processes orchestrate tissue‐level communication.

**TABLE 1:** Mechanically regulated secretion across stimulus types. Evidence level was classified as direct organelle mechanosensing when mechanical force was directly applied to an organelle membrane and elicited a defined molecular or biochemical response (e.g., channel‐mediated ion flux, protein recruitment, or enzyme/lipid activation); as indirect mechanotransduction when mechanical cues were transmitted through upstream cellular signaling pathways to regulate organelle function or secretion; and as correlative association when altered mechanical conditions were associated with a secretory phenotype, or with a change in a physical/rheological organelle property such as stiffness, without direct demonstration of the underlying molecular mechanotransduction mechanism.

Stimulus	System / Cell type	Organelle	Pathway	Secretory outcome	Evidence level	Reference
**Biaxial stretch, cell spreading**	HeLa, RPE1 cells	ER / ERES	Rac1–Sar1–COPII	↑ ER‐to‐Golgi trafficking	Indirect mechanotransduction	Phuyal et al., 2022, EMBO J. [38]
**Substrate stiffness / ECM composition**	HeLa cells	ER / ERES	FAK–SEC23A	↓ SEC23A on soft ECM	Indirect mechanotransduction	Jung et al., 2022, J. Cell Biol. [41]
**Direct force on ER tubules (LIMER)**	HeLa cells	ER membrane	TRPV1 / PKD2 channels	Ca^2^^+^ release; ↓ ER‐to‐Golgi trafficking	Direct organelle mechanosensing	Song et al., 2024, Dev. Cell [44]
**Focused ultrasound**	Invasive cancer cells (MDA‐MB‐231, PC‐3)	ER membrane	PANNEXIN‐1	Ca^2^^+^ release → cytokine/chemokine secretion	Direct organelle mechanosensing	Lee et al., 2020, Front. Cell Dev. Biol. [49]
**Cyclic stretch (6%–9%)**	Tendon cells	ER–PM contacts	STIM1	ER tension; ATP / ER stress (load‐dependent)	Indirect mechanotransduction	Chen et al., 2025, Sci. Adv. [5]
**Substrate stiffness (0.5 kPa–GPa)**	HeLa cells, fibroblasts	Golgi / TGN	Src–FAK–AMPK–GBF1	Cargo sorting: secretion vs. lysosomal degradation	Indirect mechanotransduction	Serafino et al., 2026, Adv. Sci. (Weinh) [9]
**Substrate softening / contractility inhibition**	MCF10ATk1 mammary epithelial cells	Golgi	SCAP–SREBP, Lipin‐1	↑ Lipid biosynthesis	Indirect mechanotransduction	Romani et al., 2019, Nat. Cell Biol. [52]
**Direct optical micromanipulation of Golgi membrane**	MCF10ATk1 mammary epithelial cells	Golgi membrane	DAG sensor recruitment	SCAP retention, lipid biosynthesis	Direct organelle mechanosensing	Romani et al., 2019, Nat. Cell Biol. [52]
**Substrate stiffness, stretch**	HeLa, RPE1‐ManII‐Halo cells	TGN	RAB6–PKD–CARTS	↑ TGN‐to‐PM carrier formation	Indirect mechanotransduction	Bhaskar Naidu et al., 2026, J. Cell Biol. [19]
**Optical tweezers (direct force)**	RPE‐1 cells (GFP‐Rab6)	Golgi	Actomyosin‐dependent stiffness	Organelle rigidity	Correlative association	Guet et al., 2014, Current Biology [58]
**RAB6/microtubule‐directed carrier targeting**	HeLa, RPE‐1 cells	Post‐Golgi / TGN carriers	RAB6–microtubules–ELKS	Secretion directed to focal‐adhesion‐associated exocytic hotspots	Correlative association	Fourriere et al., 2019, J. Cell Biol. [59]
**Substrate stiffness**	Hepatic stellate cells (β1 integrin‐expressing)	Plasma membrane	RhoA–caveolin‐1	↑ TIMP‐1 exocytosis	Indirect mechanotransduction	Lachowski et al., 2022, ACS Nano [32]
**Substrate stiffness (ECM rigidity)**	Preosteoblasts (β1 integrin fl/fl vs. KO)	Late endosome	β1 integrin–LAMTOR–Src–YAP	Peripheral LAMTOR/late‐endosome redistribution; promotes recycling and YAP nuclear activation	Indirect mechanotransduction	Block et al., 2020, Journal of Biological Chemistry [68]
**Substrate stiffness (1.5–28 kPa)**	COS7 cells	Secretory lysosome	VAMP7 – LRRK1 (retrograde) / VARP (anterograde)	Peripheral, readily‐releasable lysosome pool; regulated exocytosis	Indirect mechanotransduction	Wang et al., 2018, iScience [67]
**Substrate stiffness**	Hepatocellular carcinoma cells	Endolysosome / exosome	Akt–Rabin8–Rab8	↑ Exosome secretion	Indirect mechanotransduction	Wu et al., 2023, Nat. Cell Biol. [64]
**Substrate stiffness**	Mammary / cardiac fibroblasts	Endolysosome / exosome	ROCK1–c‐Myc–TRX	Biphasic EV size change	Indirect mechanotransduction	Yang et al., 2025, Small [65]
**Substrate stiffness**	Chondrocytes	Endolysosome / exosome	YAP	↑ Exosome secretion at intermediate stiffness	Indirect mechanotransduction	Lai et al., 2025, Acta Biomater. [66]
**Hypotonic stress / mechanical force (osmotic membrane stretch)**	HEK293 cells (TRPML2‐transfected); alveolar macrophages (endogenous)	Recycling endosome (Rab4A+)	TRPML2 channel	Cation channel activation; accelerates endosome tubulation/vesiculation and fast recycling	Direct organelle mechanosensing	Chen et al., 2020, Sci. Adv. [71]
**Mechanical stretch / hydrostatic pressure**	Drosophila; mouse (TMEM63A)	Late endosome / lysosome	TMEM63 channel	Mechanosensitive cation currents	Direct organelle mechanosensing	Li et al., 2024, Nat. Cell Biol. [72]
**Hydrostatic pressure**	Macrophages	Lysosome	TMEM63A → Ca^2^^+^/Na^+^ release → TFEB/TFE3	Control of lysosomal biogenesis	Direct organelle mechanosensing	Cai et al., 2024, Nat. Cell Biol. [73]

## 2 Mechanical Regulation of the ER Exit

The ER is a vast and dynamic membrane network central to protein synthesis, lipid metabolism, and calcium homeostasis [33, 34]. Secretory traffic begins here, at ER exit sites (ERES), where coat protein complex II (COPII)‐coated vesicles assemble to deliver newly synthesized cargo to the Golgi apparatus. For many years, the formation and activity of ERES were thought to depend primarily on biochemical cues such as nutrient availability and growth factor signaling [35, 36]. This view has shifted as accumulating evidence reveals that mechanical cues can regulate ER function, including ERES organization and secretory throughput. Although ER export is sensitive to mechanical cues, current evidence does not establish the ER or ERES as direct mechanosensory structures. Recent reviews have highlighted the emerging roles of intracellular organelles, including the ER, in cellular mechanosensing and mechanotransduction, while emphasizing that the molecular mechanisms underlying these responses remain incompletely defined [20, 37].

Mechanical strain has emerged as a potent regulator of ER export. Phuyal et al. (2022) demonstrated that when cells spread on micropatterned substrates or experience biaxial stretching, the number of ERES rapidly increases and ER‐to‐Golgi trafficking accelerates [38]. Surprisingly, this response requires the small GTPase Rac1, traditionally associated with actin remodeling [39], which relocalizes to the ER under mechanical load. At this unexpected location, Rac1 engages directly with Sar1, the GTPase that initiates COPII coat assembly, stabilizing it in its active conformation and sustaining COPII cycling. Structural analyses suggest the formation of Rac1–Sar1 heterodimers [40], a configuration not previously observed among small GTPases that provides a novel mechanistic layer to COPII regulation. Together, these findings define an ER‐based mechanotransduction module in which the mechanical state of the cell surface is rapidly translated into adjustments in secretory output, enabling cells to increase trafficking capacity in response to mechanical demands.

Mechanical information also reaches the ER through cell–matrix adhesion. Jung et al. (2022) demonstrated that ECM composition and focal adhesion signaling regulate the levels of COPII components [41]. Using a small interfering RNA (siRNA)‐based screen, the authors revealed that several SEC23B interactors have also been identified among components of the integrin adhesome, raising the possibility of an association between adhesion‐related signaling and the early secretory pathway; however, whether these interactions reflect direct regulation of COPII trafficking or non‐canonical functions of SEC23B remains unresolved. Indeed, cells plated on fibroblast‐derived ECM or Matrigel exhibit reduced SEC23A (Sec23 homolog A, coat complex II component) mRNA and protein levels, an effect mediated by FAK signaling. Pharmacological inhibition of focal adhesion kinase (FAK) signaling restores SEC23A abundance and rescues ER‐to‐Golgi transport defects. This adhesion‐dependent suppression of SEC23A creates a mechanochemical circuit that enables the secretory capacity of the cell to adapt to its adhesive state. This selective regulation of SEC23A is particularly relevant, as its downregulation, mediated by miR‐200 family microRNAs, remodels the secretome to favor metastatic colonization [42, 43]. Thus, ECM engagement not only influences cell shape and signaling but also directly tunes the molecular machinery that initiates secretion.

Mechanical forces act not only at ERES but also on the ER membrane itself. Song et al. (2024) developed LIMER (light‐inducible ER‐specific mechanostimulator), an optogenetic tool that applies force to ER tubules through light‐driven kinesin motors [44, 45]. Mechanical pulling of ER membranes induces rapid calcium release from the ER lumen through mechanosensitive channels, including transient receptor potential cation channel, subfamily V, member 1 (TRPV1), and polycystin‐2 (PKD2). Inhibition of these channels attenuates the calcium response, and the simultaneous inhibition of both produces an additive effect. Mechanical stimulation also inhibits ER‐to‐Golgi trafficking independently of calcium, as demonstrated by the delayed accumulation of a RUSH cargo in the Golgi, while prolonged stimulation triggers ER stress markers such as BiP upregulation and eIF2α phosphorylation [44, 46, 47]. It should be noted that LIMER applies strong, spatially localized mechanical force directly to ER tubules, and it remains unclear which physiological or pathological condition, if any, this experimental paradigm is intended to model; the observed reduction in ER‐to‐Golgi trafficking may simply reflect a direct consequence of mechanical overload on ER function, rather than a regulated cellular response. These observations indicate that mechanical stimulation of the ER can perturb ER function and transiently dampen ER‐to‐Golgi trafficking, while prolonged mechanical stress activates ER stress responses.

Rather than supporting a simple monotonic relationship between mechanical force and secretory activity, these studies suggest a context‐ and dose‐dependent response. Moderate physiological deformation can increase ER export capacity, as observed with cell spreading and biaxial stretch, whereas excessive or highly localized force can impair ER‐to‐Golgi trafficking and activate ER stress. The transition between these states may therefore reflect a mechanical window in which force is adaptive below a threshold but becomes disruptive when ER tension or load exceeds the cell's capacity for compensation. This interpretation also provides a possible framework for reconciling the apparently divergent effects reported across experimental systems, although the mechanical thresholds identified in different models cannot yet be directly compared.

The list of mechanosensitive channels operating at the ER is expanding rapidly. Piezo1, classically known for its role at the plasma membrane, has been detected at the ER where it mediates mechanosensitive calcium release and protects epidermal stem cells from DNA damage during stretch [22, 48]. Similarly, Pannexin‐1, a channel localized to both the plasma membrane and the ER, responds to focused ultrasound and can trigger calcium release from ER stores [22, 49]. The presence of multiple mechanosensitive channels may reflect specialization among ER subdomains, redundancy within the same cell type, or diversification of downstream signaling routes [50]. Together, these findings indicate that mechanical inputs can influence ER function through changes in membrane‐associated ion flux and calcium signaling, in addition to effects on trafficking machinery.

Mechanical coupling between the plasma membrane and the ER further refines these responses. Chen et al. (2025), studying tendon cells subjected to cyclic stretching, found that physiological strain increases tension at both the plasma membrane and the ER, as measured with FliptR probes. This transmission of force depends on plasma membrane–ER contact sites orchestrated by the tethering protein STIM1. Partial depletion of STIM1 abolishes the strain‐induced increase in ER tension, whereas its overexpression raises ER tension even in the absence of external stimuli. Notably, this mechanical adaptation is tightly linked to cellular energetics. Under physiological load (6% strain), ER–mitochondria contact sites expand, and ATP production rises. In contrast, excessive load (9% strain) causes pathological elevation of ER tension, leading to ER expansion, ER stress response activation, reduced ER–mitochondria contacts, and diminished ATP levels. Partial depletion of STIM1 under these overload conditions rescues these defects, highlighting that STIM1‐mediated mechanotransduction must remain within a narrow range to preserve metabolic homeostasis [5].

Together, these studies establish the ER as a mechanically regulated organelle that responds to both biochemical and physical cues to tune the earliest steps of secretion (Figure 1). Mechanical cues arising from substrate adhesion, membrane tension, cytoskeletal forces, and organelle contact sites can be transmitted to the ER, influencing calcium signaling, COPII assembly, ERES dynamics, and cellular metabolism. This regulation operates at multiple molecular levels: transcriptionally, through FAK‐mediated modulation of SEC23A expression in response to ECM engagement, and post‐translationally, through direct protein interactions such as Rac1–Sar1 heterodimerization, STIM1‐mediated contact sites, and activation of mechanosensitive calcium channels that rapidly alter ER function without requiring new gene expression. Through these complementary transcriptional and post‐translational mechanisms, the ER adjusts secretory capacity to match the mechanical demands and constraints of the cell microenvironment.

**FIGURE 1:**
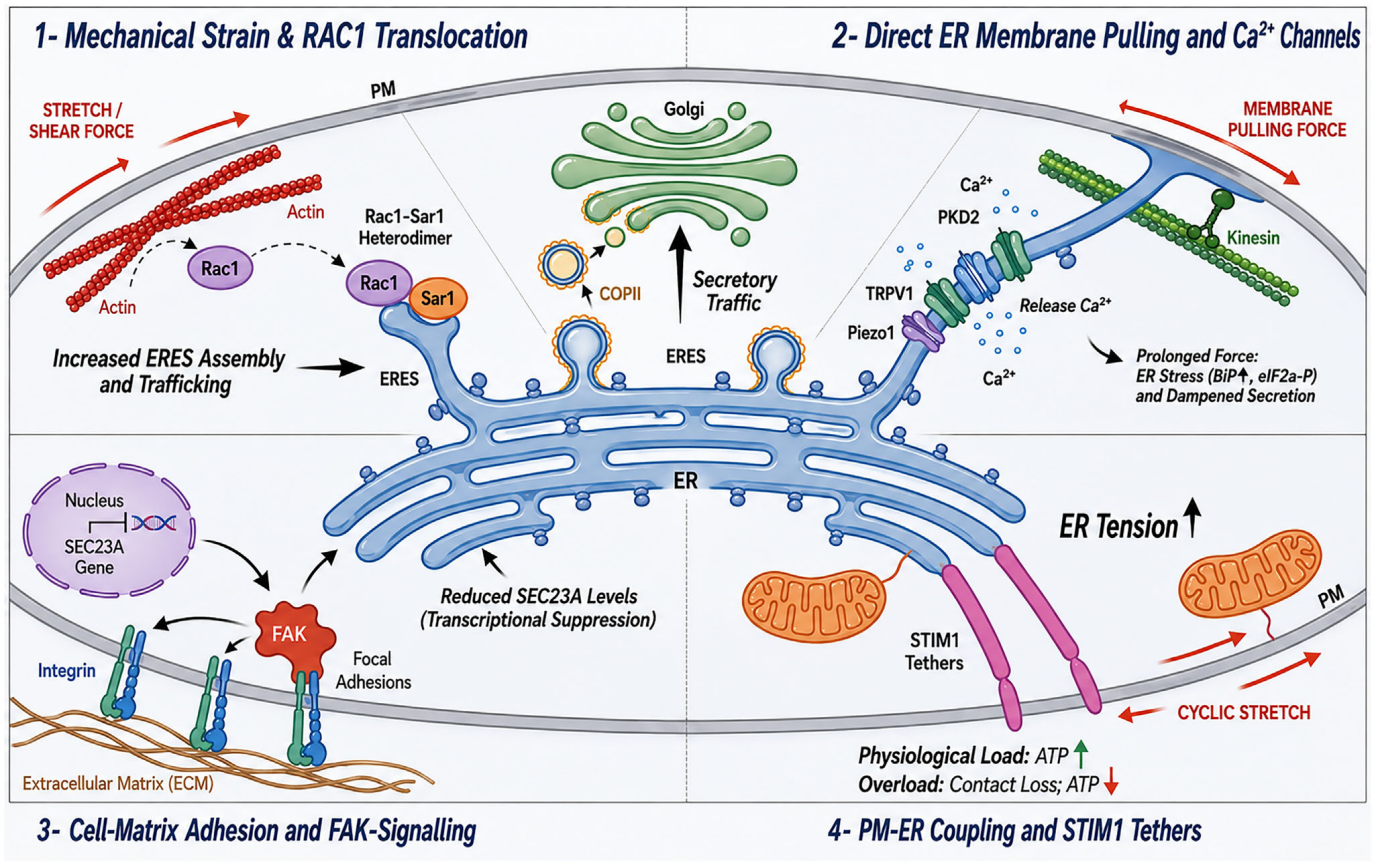
Mechanically regulated ER‐Exit: integrating physical cues and secretion. The ER integrates mechanical signals from the ECM and plasma membrane to regulate COPII trafficking via complementary mechanisms. (1) Mechanical strain and Rac1‐COPII regulation: cell spreading and biaxial stretching relocalize Rac1 to the ER, where it stabilizes active Sar1, increasing ERES number and accelerating ER‐to‐Golgi trafficking. (2) Mechanosensitive Ca^2^^+^ channels: membrane tension on ER tubules activates TRPV1, PKD2, and Piezo1, triggering luminal Ca^2^^+^ release. Excessive load also inhibits ER‐to‐Golgi transport and induces ER stress (BiP upregulation, eIF2α phosphorylation). (3) ECM stiffness and focal adhesion signaling: integrin engagement activates FAK, suppressing miR‐200‐mediated SEC23A downregulation and adjusting secretory capacity and secretome composition to the cell's adhesive state. (4) STIM1‐mediated plasma membrane–ER coupling: physiological strain (6% stretch) raises plasma membrane /ER tension via STIM1 contact sites, expanding ER–mitochondria contacts and boosting ATP. Overload (9% strain) instead causes pathological ER tension, expansion, stress activation, and reduced ATP, rescued by partial STIM1 depletion. Together, these pathways define the ER as a mechanoresponsive hub translating substrate stiffness, cytoskeletal forces, and contact‐site geometry into rapid, post‐translational secretory adjustments. Sec23/24 coat subunits; dashed arrows indicate indirect/proposed connections.

## 3 Mechanical Regulation of the Golgi Apparatus

The Golgi apparatus occupies a central position in the secretory pathway, receiving cargo from the ER, processing proteins, and sorting them at the TGN for delivery to the plasma membrane, endosomes, or lysosomes. Historically viewed through a biochemical lens, cargo sorting has been attributed to molecular recognition signals and adaptor proteins [15, 51]. However, emerging evidence reveals that the Golgi is subject to extensive mechanical regulation, tuning secretory output in response to physical cues and influencing whether cargo proceeds toward secretion or is diverted to degradation [9]. The concept that the Golgi apparatus actively integrates mechanical cues was first substantiated by Romani et al. (2019), who demonstrated that ECM stiffness directly regulates Golgi‐dependent trafficking and lipid metabolism. In this study, mechanical relaxation of the cellular environment, achieved either by culturing cells on soft substrates or by pharmacological inhibition of actomyosin contractility, resulted in alterations in Golgi function. Specifically, reduced mechanical tension led to retention at the Golgi of SCAP (SREBP‐cleavage–activating protein), the chaperone of sterol regulatory element‐binding protein (SREBP), thereby promoting SREBP activation and driving lipid biosynthesis independently of canonical mechanotransduction pathways such as YAP/TAZ, mTOR, or AMP‐activated protein kinase (AMPK). Mechanistically, these effects were linked to changes in Golgi membrane tension and diacylglycerol (DAG) availability, implicating the Golgi membrane itself as a mechanically responsive platform, with direct evidence for organelle‐level mechanosensing provided by optical micromanipulation experiments [52]. Together, these findings established the Golgi as a central hub where mechanical inputs are translated into metabolic and trafficking outputs, providing a conceptual framework for subsequent studies addressing how physical properties of the microenvironment influence secretory pathway organization and function.

Increasing evidence from multiple studies indicates that mechanical cues can regulate Golgi organization and function through distinct signaling and membrane‐associated mechanisms. In this context, our recent study identified a mechanically regulated sorting mechanism operating at the Golgi that links ECM stiffness to post‐Golgi cargo fate [9]. Tracking synchronized transport of multiple cargoes, including hGH‐FM2‐GFP, TIMP1, TIMP2, and procollagen I, across substrates of defined rigidity (0.5 kPa, 8 kPa, and >GPa), we discovered that on stiff substrates these proteins efficiently progressed through the Golgi and were secreted, whereas on soft substrates they transited normally through the Golgi compartments but were rapidly rerouted to LAMP1‐positive lysosomes after exiting the Golgi, resulting in degradation and reduced secretion. Critically, this sorting switch establishes the Golgi as a mechanoresponsive gatekeeper adjusting the balance between secretion and degradation in response to tissue stiffness.

Our recent work showed that ECM stiffness regulates post‐Golgi cargo fate through a Src–FAK–AMPK–GBF1 (Golgi brefeldin A‐resistant guanine nucleotide exchange factor 1) signaling axis, providing evidence that mechanical information can influence Golgi‐dependent sorting. The tyrosine kinases FAK and Src exhibited robust phosphorylation on stiff substrates and reduced activity on soft matrices, with functional experiments revealing divergent roles: Src depletion impaired cargo export from the Golgi, while FAK depletion redirected cargo to lysosomes, phenocopying soft substrate conditions. Phosphoproteomic screening identified GBF1, a guanine nucleotide exchange factor essential for Arf1 activation, as the key mechanically regulated node, with serine 1318 (S1318) phosphorylated under soft conditions and unphosphorylated under stiff conditions. Expression of a phosphodeficient GBF1 S1318A mutant in cells on soft substrates fully restored secretion and prevented lysosomal targeting, while the same mutant rescued secretion in FAK‐depleted cells on stiff substrates, establishing GBF1 phosphorylation at S1318 as the molecular switch determining post‐Golgi cargo fate. In this context, AMPK, a master regulator of cellular energy homeostasis, was identified as the kinase responsible for S1318 phosphorylation. AMPK was highly phosphorylated on soft substrates, and its silencing rescued secretion under soft conditions. Conversely, activating AMPK on stiff substrates through glucose starvation or metformin treatment reduced secretion and promoted lysosomal degradation. Expression of the GBF1 S1318A mutant bypassed AMPK‐dependent inhibition, and co‐immunoprecipitation confirmed physical interaction between AMPK and GBF1. These findings establish a signaling pathway in which ECM stiffness regulates Golgi sorting through the Src–FAK–AMPK–GBF1 axis: on stiff substrates, integrin engagement activates Src and FAK, suppressing AMPK and preventing GBF1 phosphorylation, thereby favoring secretion; conversely, on soft substrates, reduced Src/FAK signaling permits AMPK activation, leading to GBF1 phosphorylation, and diversion of cargo to lysosomes [9]. To note, Src also directly phosphorylates GBF1 on ten tyrosine residues, promoting GBF1‐dependent retrograde trafficking of Golgi enzymes to the ER through tubular carriers [53]. Because anterograde cargo trafficking and retrograde transport are functionally coupled processes required for Golgi homeostasis, one possibility is that site‐specific phosphorylation of GBF1 by these distinct kinase pathways may contribute to coordinating anterograde and retrograde trafficking according to the cellular signaling and mechanical context. Whether these pathways converge mechanistically or operate independently remains to be determined. This mechanism should be considered alongside independent studies showing that mechanical cues regulate Golgi morphology, trafficking, lipid composition, and secretory function through distinct pathways [19, 32, 52, 54, 55, 56, 57, 58, 59]. Together, these findings support a broader model of mechanical regulation of the Golgi, rather than a mechanism restricted to the pathway identified by our group.

AMPK‐mediated GBF1 regulation extends to specialized secretory organelles: in endothelial cell monolayers, AMPK activation modulates both the release kinetics and morphology of Weibel‐Palade bodies, thus linking mechanical and metabolic inputs to stimulus‐dependent exocytosis [54]. This AMPK‐GBF1 axis integrates mechanical inputs with metabolic status, redirecting secretory resources toward degradation and recycling. The convergence of mechanotransduction and energy sensing at the Golgi therefore might link tissue mechanics to cellular metabolism and secretome composition.

GBF1 also intersects with cell‐cycle regulation, further reinforcing this integration of mechanics, metabolism, and Golgi function. During mitosis, cytoskeletal reorganization and changes in cell shape trigger GBF1 phosphorylation through multiple pathways. Mao et al. (2013) demonstrated that at mitotic entry, AMPK phosphorylates GBF1 at threonine 1337, causing its dissociation from Golgi membranes and reducing Arf1‐GTP levels, thereby promoting Golgi fragmentation required for orderly mitotic progression [55]. Magliozzi et al. (2018) further showed that during cytokinesis, GBF1 is phosphorylated on serine 292 and serine 297 by CK2, activated downstream of CDK1/cyclin B. These modifications target GBF1 for degradation via the SCFβTrCP ligase, which enables Golgi twin migration, fusion, and proper ribbon reassembly at mitotic exit. Stabilization of GBF1 disrupts this cycle, leaving the Golgi fragmented and preventing resumption of secretion [56]. These findings underscore how mechanical changes and kinase‐driven cues intersect to orchestrate Golgi disassembly and reassembly through the cell cycle, with GBF1 serving as a central regulatory node. A key unresolved question is whether these sites act independently, hierarchically, or combinatorially, and whether their effects reflect changes in GBF1 localization, enzymatic activity, stability, or interaction with specific trafficking partners.

Mechanical regulation also affects the metabolic functions of the Golgi apparatus, further extending the link between tissue stiffness and cellular energetics. Romani et al. (2019) demonstrated that ECM stiffness influences lipid metabolism through Golgi‐dependent trafficking pathways. Cells cultured on soft ECM, or subjected to inhibition of contractility, accumulated triglycerides, ceramides, cholesterol, and DAG, resulting from activation of SREBP transcription factors, the master regulators of lipid biosynthesis. Briefly, the mechanical trigger (reduced contractility) leads to retention of SCAP at the Golgi, where it enables the proteolytic processing and release of active SREBP, which subsequently translocates to the nucleus. This pathway operates independently of mTOR, AMPK, or YAP/TAZ. Notably, lipin‐1, a phosphatide phosphatase enriched at the Golgi, emerged as a key mediator of this process, as its inhibition phenocopied soft‐ECM conditions by preventing SCAP retrieval to the ER and promoting SREBP activation. Lipin‐1 regulation of DAG at the Golgi also links membrane lipid composition directly to Arf1 recruitment and coat protein complex I (COPI) formation [52]. This lipid‐based mechanotransduction mechanism positions the Golgi as a deformable organelle whose membrane mechanics respond to contractile states, modulating metabolic outputs accordingly. As highlighted by Théry & Pende (2019), the discovery that actomyosin contractility directly impacts Golgi mechanics and lipid metabolism through a Lipin‐1‐dependent DAG regulation represents a conceptual advance in mechanobiology, expanding the concept of mechanically responsive structures beyond focal adhesions and the nucleus to include the endomembrane system, although in this case mechanical information reaches the Golgi indirectly, through cytoskeletal contractility, rather than via direct force sensing by the organelle. Implications of this discovery extend to diverse physiological contexts, such as muscle contraction and stem cell differentiation. In skeletal muscle, the relationship between contraction frequency and oxidative metabolism may be partially mediated by this mechanism, while in mesenchymal stem cells, mechanical relaxation that promotes adipogenic differentiation likely involves SREBP activation downstream of reduced contractility. Furthermore, Lipin‐1 deficiency in humans causes rhabdomyolysis and metabolic myopathy with lipid accumulation, underscoring the physiological relevance of this mechanically regulated pathway [52, 57].

Mechanically softened Golgi membranes, generated using optical micromanipulation, resulted in SCAP retention at the Golgi and therefore lipid biosynthesis. Conversely, direct application of mechanical force increased the recruitment of a DAG sensor, confirming the intrinsic mechanosensitivity of the Golgi membrane. The relevance of this pathway extends to both physiology and pathology: in human pluripotent stem cells, Rho‐associated coiled‐coil containing protein kinase (ROCK) inhibition promotes cell survival through this pathway, whereas in keloids, characterized by abnormally stiff ECM, SREBP target genes are downregulated [52, 57].

The discovery of stiffness‐dependent Golgi sorting has implications for diseases characterized by aberrant ECM remodeling. In fibrosis and desmoplastic tumors, elevated ECM rigidity activates Src and FAK, suppresses AMPK, and prevents GBF1 phosphorylation, thereby favoring secretion of ECM components such as collagen and matrix crosslinking enzymes over their degradation. The secreted proteins further stiffen the matrix, creating a pathological positive‐feedback loop that could be therapeutically targeted by manipulating GBF1 phosphorylation or AMPK activity [9]. Mechanistically, this model is supported by the evidence that GBF1 localizes to the trans‐Golgi network (TGN), where its substrate Arf1 is organized into two distinct clathrin‐coated membrane domains associated with different trafficking routes. One domain directs cargo toward the plasma membrane, whereas the other promotes endocytic recycling [60]. These findings suggest that GBF1 phosphorylation may regulate cargo fate by selectively controlling Arf1 activity within specific TGN subdomains.

Mechanical forces also regulate a distinct step of Golgi export. Independently of the Src‐FAK‐AMPK‐GBF1 sorting mechanism described above, which determines whether cargo is directed toward secretion or lysosomal degradation, Naidu et al. [19] demonstrated that the Golgi adjusts the efficiency of anterograde carrier biogenesis in response to mechanical cues. Using live‐cell imaging and RUSH assays, they showed that mechanical stimuli such as cell spreading, substrate stiffness, and equiaxial strain enhance the formation of CARTS (CARriers of the TGN to the cell Surface), RAB6‐ and protein kinase D (PKD)‐dependent carriers mediating TGN‐to‐plasma membrane transport. Cells cultured on stiff fibronectin or subjected to mechanical stretching generated more CARTS than cells grown on soft substrates or poly‐L‐lysine, which prevents integrin‐mediated adhesion. Notably, CARTS preferentially fused at exocytic hotspots adjacent to focal adhesions, highlighting a spatial link between adhesion sites and TGN export. Using Halo‐Flipper probes with fluorescence lifetime imaging microscopy (FLIM), Naidu et al. (2026) [19] provided direct evidence that mechanical stretching or increased adhesion raises Golgi membrane tension, whereas soft substrates or actin depolymerization reduce it. These tension shifts correlated with alterations in DAG, a key PKD activator at the TGN, with DAG abundance increasing under stiff substrates, thereby providing a biochemical link between membrane tension and carrier release [19].

The mechanical responsiveness of the Golgi extends to its physical properties. Guet et al. (2014) used optical tweezers to measure organelle stiffness and found the Golgi to be markedly more rigid than the surrounding cytoplasm, and that its stiffness is dynamically controlled by the cytoskeleton: actin depolymerization softens the Golgi, while enhanced actomyosin contractility increases its rigidity [58]. Mechanical forces acting on the Golgi influence the generation of Rab6‐positive carriers, suggesting a link between cytoskeletal tension and post‐Golgi trafficking; however, because this evidence derives from measurements of passive organelle stiffness rather than from a demonstrated force‐elicited molecular response, it should be regarded as a correlative association rather than direct organelle mechanosensing. One mechanism connecting mechanical force to secretory export involves microtubule acetylation: mechanical inputs increase microtubule acetylation, thereby promoting CARTS formation. Consistently, pharmacological induction of microtubule acetylation with the histone deacetylase 6 (HDAC6) inhibitor tubacin enhanced CARTS formation even in the absence of external force. However, tubacin did not alter membrane tension or DAG levels, suggesting that microtubule acetylation primes secretory export but does not replace force‐dependent PKD activation. Together these findings support a model in which microtubule acetylation, RhoA activation, PLCε‐dependent DAG production, and PKD‐mediated fission form a coordinated set of inputs that specifically regulate carrier biogenesis at the Golgi [19].

Mechanical regulation continues beyond the Golgi, influencing where post‐Golgi carriers fuse with the plasma membrane. Fourriere et al. (2019) [59] showed that microtubules and RAB6‐positive carriers direct secretion toward focal adhesions, creating exocytic hotspots enriched at sites of high mechanical engagement. This concept was further extended by Naidu et al. (2026) [19], who demonstrated that CARTS preferentially fuse at adhesion‐associated hotspots and that this process is enhanced by substrate stiffness and cell spreading. Cytoskeletal dynamics integrate seamlessly: the Rho–mDia1 pathway promotes formation of Rab6‐positive carriers, linking actin polymerization directly to post‐Golgi trafficking [61]. Mechanotransduction‐based regulation also occurs at the plasma membrane. Lachowski et al. (2022) showed that substrate stiffening elevates membrane tension via a β1 integrin–RhoA pathway, promoting caveolae formation and accelerating TIMP‐1 exocytosis through caveolin‐1 and dynamin‐2, positioning membrane tension as a rapid, post‐translational regulator of secretory flux [32].

Taken together, these studies suggest that mechanical information may be decoded at the Golgi through partially distinct but potentially convergent layers of regulation (Figure 2). Romani et al. [52] linked reduced contractility to Golgi membrane mechanics and DAG‐dependent control of SCAP retrieval and lipid metabolism, whereas Naidu et al. [9, 19] connected increased Golgi tension and DAG production to PKD‐dependent CARTS formation. Our findings identify a complementary signaling layer in which substrate mechanics are translated through Src–FAK–AMPK signaling into GBF1 phosphorylation and a change in post‐Golgi cargo fate [9, 19]. Thus, mechanical regulation at the Golgi may not represent a single linear pathway but rather a distributed system in which membrane physical state, kinase activity, and trafficking machinery independently modulate distinct aspects of secretory output. Whether these mechanisms are functionally coupled, for example, through shared regulation of DAG, Arf1/GBF1 activity, or distinct cargos, their pools and availability, or instead operate in parallel remains an open question.

**FIGURE 2:**
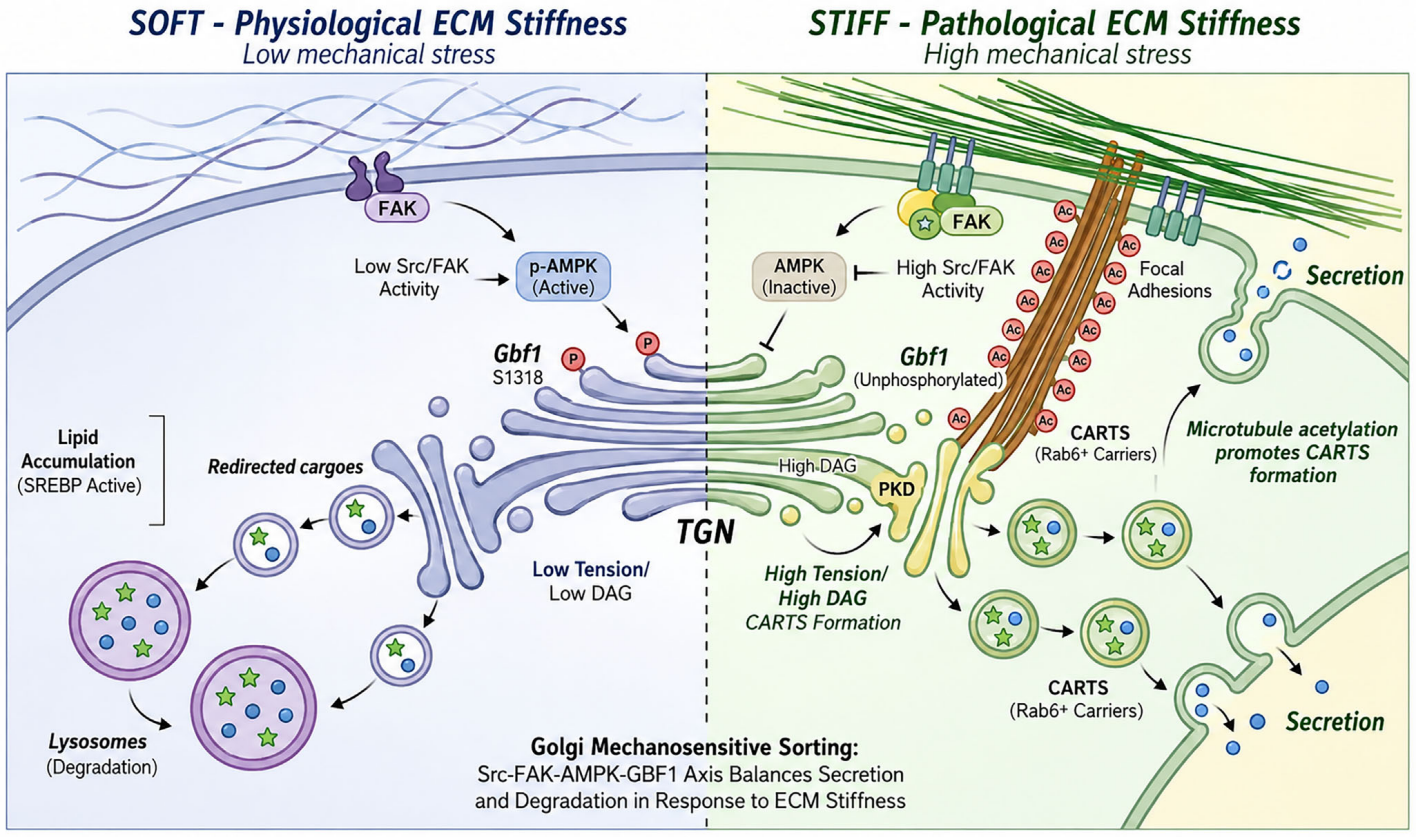
Mechanically regulated sorting at the Golgi apparatus. (Left panel; soft ECM, low mechanical stress). On compliant substrates, reduced integrin engagement lowers Src and FAK activity, releasing tonic AMPK suppression. Active AMPK phosphorylates GBF1 at serine 1318 (GBF1‐pS1318), impairing its GEF activity toward Arf1 at the TGN. Consequently, post‐Golgi carrier biogenesis is reduced: low TGN membrane tension and DAG levels limit PKD‐dependent fission and CARTS (RAB6‐positive carriers) formation. Secretory cargoes, including TIMP1, TIMP2, hGH, and procollagen I, are redirected after TGN exit to LAMP1‐positive lysosomes for degradation rather than secretion. Reduced contractility also retains SCAP at the Golgi, promoting SREBP activation, and lipid accumulation. Thus, soft substrates suppress secretion while favoring catabolism and lipid biosynthesis. (Right panel; stiff ECM, high mechanical stress). On rigid substrates, integrin clustering at focal adhesions activates Src and FAK, suppressing AMPK and maintaining GBF1 in its active, unphosphorylated state. Active GBF1 sustains Arf1‐GTP at the TGN, promoting membrane tension and DAG production via PLCε and RhoA. DAG activates PKD, driving efficient CARTS fission and TGN‐to‐plasma‐membrane transport. Acetylated microtubules (Ac), enriched on stiff substrates, further promote CARTS formation and directional delivery. Secretory cargoes are efficiently delivered to the cell surface, supporting ECM components, signaling factors, and matrix‐remodeling enzymes. Coupling of CARTS fusion to focal adhesions spatially concentrates newly secreted cargo, reinforcing ECM stiffening in pathological contexts such as fibrosis and desmoplastic tumors. Solid arrows indicate direct, experimentally validated relationships. Blunt‐ended lines (⊣) indicate inhibitory relationships. Dashed arrows indicate indirect or multi‐step connections. Stars (☆) represent secretory cargo proteins tracked in synchronized transport assays. Blue dots represent secreted factors released at the plasma membrane. The dashed vertical line separates soft (left) and stiff (right) conditions within a single cell diagram centered on the TGN. Abbreviations: ECM, extracellular matrix; TGN, trans‐Golgi network; GEF, guanine nucleotide exchange factor; DAG, diacylglycerol; CARTS, carriers of the TGN to the cell surface; SCAP, SREBP‐cleavage–activating protein; SREBP, sterol regulatory element‐binding protein; PKD, protein kinase D; Ac, acetylated microtubule; AMPK, AMP‐activated protein kinase; and FAK, focal adhesion kinase.

In summary, Golgi has emerged as a critical, mechanically regulated compartment that translates physical cues into secretory decisions, maintaining tissue homeostasis under physiological conditions and driving pathology when dysregulated in fibrosis and cancer.

## 4 Extracellular Vesicles: the Mechanically Regulated Arm of the Secretome

Cells do not rely solely on the classical secretion of soluble factors and membrane proteins to communicate; they also release EVs, including exosomes and microvesicles, which act as pivotal mediators of intercellular signaling [62, 63]. Emerging evidence indicates that EV secretion is highly responsive to mechanical cues, revealing an additional layer of mechanical regulation over the secretome. In particular, ECM stiffness influences the quantity and composition of exosomes. Wu et al. (2023) showed that hepatocellular carcinoma cells, as well as other cancer cell types, release significantly more exosomes when cultured on stiff ECM substrates compared with soft ones. This stiffness‐dependent increase, confirmed by nanoparticle tracking analysis and electron microscopy, was accompanied by elevated levels of canonical exosome markers, including CD63, Alix, Tsg101, and Syntenin [64]. Beyond cancer cells, substrate stiffness also regulates EV size and secretion in stromal cells. Yang et al. [65] demonstrated that mouse mammary and cardiac fibroblasts cultured on substrates of varying stiffness (1–60 kPa) secreted EVs that exhibited biphasic changes in size: EV diameter increased on substrates below 20 kPa, peaking at approximately 309 nm for mammary fibroblasts and 191 nm for cardiac fibroblasts at 20 kPa, and decreased on stiffer substrates (>20 kPa). These changes occurred without significant changes in EV concentration or surface marker expression (CD9, CD81). Similarly, Lai et al. [66] found that chondrocytes cultured on GelMA hydrogels mimicking normal cartilage stiffness (65.08 kPa) secreted approximately three times more exosomes than those on substrates mimicking osteoarthritic stiffness (18.85 kPa), demonstrating an optimal mechanical threshold for exosome production. Interestingly, premalignant and tumorigenic cells displayed a greater sensitivity to ECM stiffness than nonmalignant counterparts, suggesting that oncogenic mutations amplify the mechanical regulation of secretion [64]. Mechanistically, this response is mediated by an Akt–Rabin8–Rab8 signaling axis in cancer cells: stiff ECM enhances Akt phosphorylation, which in turn phosphorylates the guanine nucleotide exchange factor Rabin8 at Ser149, thereby promoting Rab8 activation and vesicular trafficking. In fibroblasts, mechanotransduction pathways involving ROCK1, myosin II, p53, and thioredoxin (TRX) regulate stiffness‐dependent EV secretion, and inhibition of either myosin II or ROCK abolishes the stiffness‐induced EV size changes. Stiffness‐mediated EV alterations in mammary fibroblasts operate primarily through the ROCK1–c‐Myc–TRX axis, whereas cardiac fibroblasts rely on both TRX signaling on stiff substrates and p53‐mediated pathways on soft substrates [65]. In chondrocytes, substrate stiffness regulates exosome secretion through YAP‐dependent signaling, with substrates of intermediate stiffness promoting YAP nuclear localization and activation [66]. Modulation of any component within these pathways directly affects exosome release, highlighting the presence of tightly regulated mechanically responsive signaling circuits [64, 65, 66]. Thus, the mechanical regulation of EVs appears to depend not only on the magnitude of the mechanical stimulus but also on cell identity, the specific EV property measured, and the mechanical range explored. These differences caution against interpreting “stiffness‐dependent EV secretion” as a generalizable increase in vesicle output and instead suggest that mechanical cues may tune distinct quantitative and qualitative features of EV biology.

The positioning of endolysosomal compartments is also regulated by mechanical cues. ECM stiffness controls the distribution of secretory lysosomes through the coordinated action of motor proteins, with LRRK1 driving retrograde movement and VARP controlling anterograde transport, creating a mechanical “tug‐of‐war” that determines lysosome localization [22, 67]. Proper lysosomal positioning is essential for cells to adjust their internal mechanics in response to ECM rigidity, underscoring the importance of mechanically regulated exocytosis in cellular adaptation. Similarly, the intracellular distribution of late endosome is modulated through the mechanically regulated redistribution of the LAMTOR complex, a key endolysosomal signaling hub [22, 68]. Beyond these intracellular pathways, additional mechanical factors such as plasma membrane tension and caveolae dynamics may influence EV biogenesis. Caveolae, by buffering membrane tension and organizing trafficking routes, potentially act as mechanosensitive platforms for vesicle budding and cargo sorting, integrating plasma membrane mechanics with Golgi‐ and TGN‐based secretory processes [32].

The functional consequences of this mechanically tuned EV secretion are profound. Exosomes produced under stiff ECM conditions are not only more abundant but carry distinct cargo, including the Notch ligand Jagged1, which can directly impact recipient cells. Proteomic analysis revealed that substrate stiffness alters EV protein composition, with differential packaging of redox‐associated proteins including thioredoxin, thioredoxin‐dependent peroxidase reductase, and peroxiredoxin‐5 in fibroblast‐derived EVs [65]. The biological impact of stiffness‐tuned exosomes extends across diverse pathological contexts. In osteoarthritis models, exosomes from medium stiffness substrates (ExoMedium), mimicking healthy cartilage, enhanced chondrocyte proliferation, and migration while reducing inflammatory markers (matrix metalloproteinase 13 [MMP13], interleukin‐6 [IL‐6]), and promoting ECM components (collagen II, aggrecan). Conversely, exosomes from soft substrates (ExoSoft), resembling osteoarthritic tissue, exacerbated inflammation, and matrix degradation. Critically, ExoMedium suppressed the pro‐inflammatory M1 macrophage phenotype (CD86+) and promoted anti‐inflammatory M2 phenotype (CD206+), whereas ExoSoft enhanced M1 polarization with a 45‐fold increase in IL‐6 expression, demonstrating that matrix stiffness orchestrates paracrine immune modulation through exosome‐mediated intercellular communication [66]. In mouse models, injections of exosomes derived from stiff ECM cultures resulted in larger tumors, elevated proliferation markers, and increased Notch signaling activity compared to exosomes from soft ECM conditions [64, 69, 70]. Analysis of patient liver tissues revealed a correlation between Notch activation and poor clinical outcomes, including liver damage, cirrhosis, elevated alanine transaminase, reduced albumin levels, and signatures of hepatic stellate cell activation [64]. These observations indicate that ECM stiffness does more than modulate exosome quantity; it shapes the composition and functional impact of vesicle cargo, establishing a mechanically driven paracrine signaling network (Figure 3). This mechanically regulated EV pathway, mediated through indirect mechanotransduction cascades (Akt–Rabin8–Rab8, ROCK1–myosin II–p53–TRX, and YAP signaling) rather than through direct mechanosensing by the vesicle‐forming membranes themselves, thus constitutes a critical link between the physical microenvironment and intercellular communication, with significant implications for tumor progression and cellular adaptation to mechanical cues across diverse pathological contexts including fibrosis, cancer, cardiovascular disease, and osteoarthritis.

**FIGURE 3:**
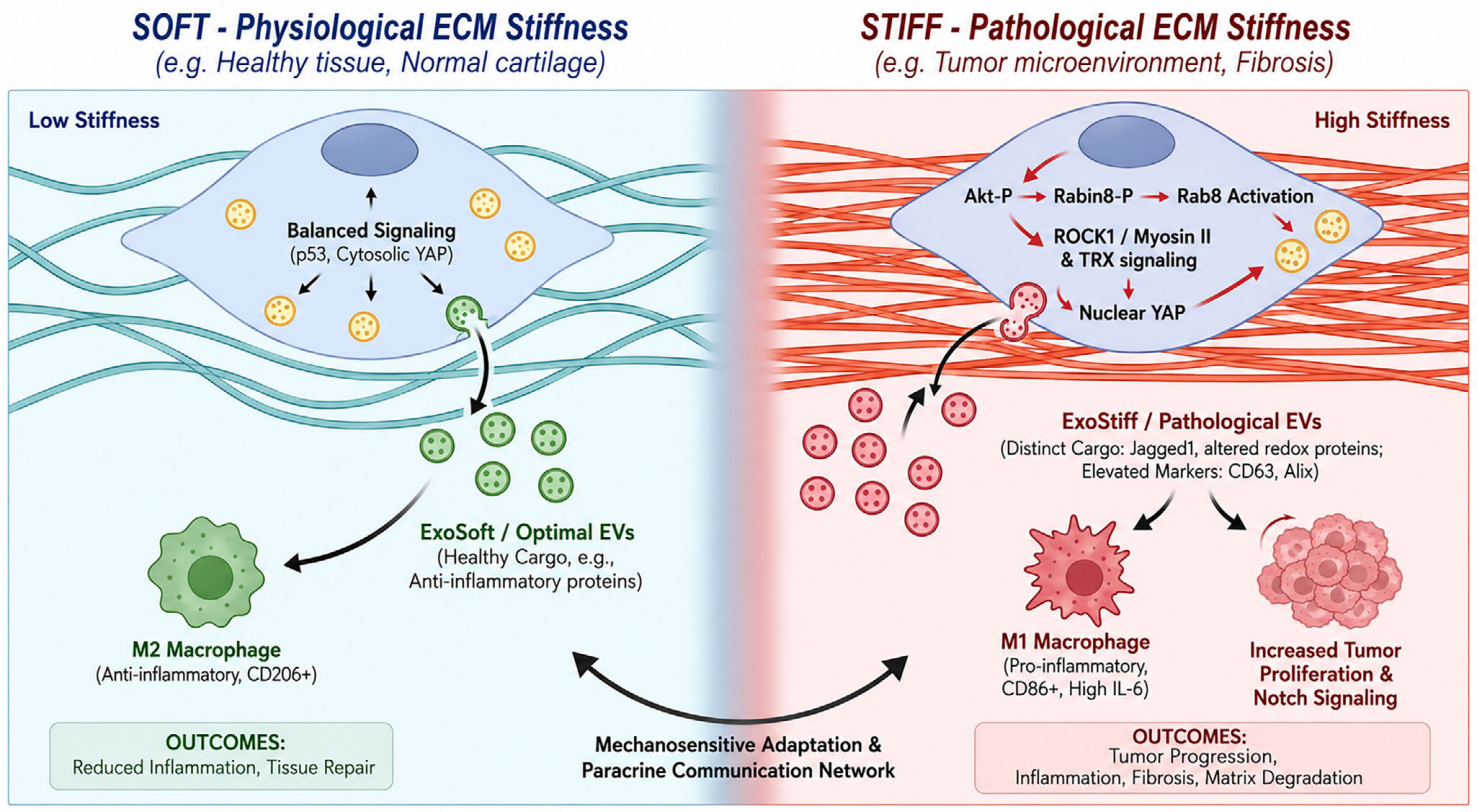
EVs as mechanically regulated mediators of intercellular communication. (Left panel; soft‐physiological ECM Stiffness, e.g., healthy tissue, normal cartilage). On compliant substrates mimicking physiological mechanics, cells exhibit balanced mechanotransduction with p53 activity, and cytosolic (inactive) YAP retention. EV biogenesis remains moderate, generating ExoSoft/Optimal EVs enriched in anti‐inflammatory cargo. In mammary and cardiac fibroblasts, soft‐substrate EVs are regulated partly through p53‐dependent pathways and promote anti‐inflammatory M2 macrophage polarization (CD206+), supporting tissue repair. In cartilage models, exosomes from medium‐stiffness substrates enhance chondrocyte proliferation and migration, reduce MMP13 and IL‐6, and promote collagen II and aggrecan, supporting tissue homeostasis. (Right panel; stiff‐pathological ECM Stiffness, e.g., tumor microenvironment, fibrosis). On rigid substrates, three mechanosensitive pathways converge to enhance EV secretion. In cancer cells, stiffness increases Akt phosphorylation, activating Rabin8 at Ser149 and promoting Rab8‐dependent trafficking. In fibroblasts, ROCK1–Myosin II activation drives EV biogenesis through the ROCK1–c‐Myc–thioredoxin (TRX) axis, with cardiac fibroblasts also engaging TRX‐independent pathways. Stiff ECM also promotes YAP nuclear activation, which regulates exosome secretion in chondrocytes. These pathways generate ExoStiff/Pathological EVs with distinct cargo, including Jagged1, altered redox proteins, and elevated CD63, Alix, Tsg101, and Syntenin‐1. ExoStiff EVs promote pro‐inflammatory M1 macrophage polarization (CD86+, increased IL‐6), tumor proliferation, and Notch signaling, and progressive ECM stiffening in fibrosis, establishing a pathological positive‐feedback loop. (Centre; mechanically responsive adaptation and paracrine communication network). Bidirectional arrows indicate that EV‐mediated communication is reciprocal: recipient‐cell reprogramming feeds back to remodel the mechanical microenvironment, reinforcing either homeostatic (left) or pathological (right) states. Solid arrows indicate direct, experimentally validated relationships. Yellow organelles represent multivesicular bodies (MVBs)/late endosomes en route to EV secretion. Green EVs (left) denote physiological exosomes; red/pink EVs (right) denote pathological exosomes. Abbreviations: ECM, extracellular matrix; EV, extracellular vesicle; MVB, multivesicular body; TRX, thioredoxin; YAP, Yes‐associated protein; ROCK1, Rho‐associated coiled‐coil containing protein kinase 1; GEF, guanine nucleotide exchange factor; MMP13, matrix metalloproteinase 13; IL‐6, interleukin‐6; CD206, mannose receptor (M2 marker); and CD86, co‐stimulatory molecule (M1 marker).

## 5 Future Directions and Open Questions

Despite significant advances, many fundamental questions remain unresolved regarding the mechanisms and physiological relevance of mechanosensitive secretion. These questions span multiple scales of biological organization, from the molecular identity of mechanosensors to the translational challenges of implementing mechanotherapy in clinical practice.

At the molecular level, identifying the direct mechanosensors embedded in organelle membranes represents one of the most pressing challenges and is echoed as an open question in recent organelle‐focused reviews [20, 37]. While TRPV1 and PKD2 have been implicated in sensing ER membrane tension [44], and Golgi membranes themselves appear capable of responding to mechanical stress [52], the precise molecular sensors remain incompletely characterized. It remains unclear whether organelles possess dedicated Piezo‐like channels or whether alternative mechanisms, such as lipid raft organization or curvature‐induced stress, serve as mechanosensory elements. Extending this question across the endomembrane system reveals significant gaps in our knowledge. While mechanosensitive ion channels have been identified in the ER and endolysosomes, their presence and function in other secretory organelles remain largely unexplored [22]. In endolysosomes, TRPML2 responds to hypotonic stress and is essential for recycling endosome function [71], while TMEM63 proteins mediate mechanosensitive cation currents in late endosomes and lysosomes [72]. TMEM63A, a member of the TMEM63 protein family, is activated by hydrostatic pressure on lysosomes in macrophages, leading to calcium and sodium release that controls lysosomal biogenesis through TFEB and TFE3 activation [22, 73]. These findings raise intriguing questions about whether the Golgi apparatus or ERES harbor mechanosensitive ion channels, and if so, whether these channels contribute to the rapid, post‐translational regulation of trafficking observed under mechanical stress. Identifying such channels and characterizing their roles could reveal an entirely new layer of mechanosensitive control over secretion.

Equally critical is understanding the transmission of mechanical forces through the cytoplasm to reach these intracellular sensors. A fundamental question concerns whether different forms of mechanical forces, such as stretch, compression, and shear stress, activate distinct mechanosensors or converge on shared signaling nodes [22]. Most studies focus on substrate stiffness or biaxial stretch, yet cells within tissues experience complex, dynamic mechanical environments. Understanding whether different force modalities differentially affect secretion will be essential for building an integrative framework. The relative contributions of actin filaments, microtubules, and intermediate filaments to force transmission toward intracellular organelles remain to be systematically defined [22]. The existence of specialized cytoskeletal tracks or organelle–cytoskeleton attachment sites that act as mechanical conduits represents an intriguing possibility that warrants further investigation. While membrane contact sites such as STIM1 link the plasma membrane to the ER and mediate mechanical coupling [5], the pathways connecting mechanical inputs to the Golgi, endosomes, and lysosomes are less well understood.

Understanding how cells generate cargo‐specific responses to mechanical stimuli adds another layer of complexity. Serafino et al. (2026) demonstrated that mechanical inputs regulate the balance between secretory and degradative sorting, yet the signals that determine cargo‐specific outcomes remain unknown [9]. It is unclear whether certain cargoes possess intrinsic mechanosensitive motifs or whether cytoplasmic adaptors mediate this specificity. Furthermore, how luminal cargoes, lacking cytoplasmic domains, are differentially sorted in response to mechanical cues remains an open question that challenges our current models of mechanotransduction.

The temporal and spatial dynamics of mechanosensitive secretion represent another frontier requiring systematic investigation. Mechanical sensing at focal adhesions, cytoskeletal rearrangement, organelle repositioning, membrane tension fluctuations, and vesicular trafficking occur across timescales ranging from seconds to hours. Mapping the precise temporal sequence of these events would help clarify causal relationships between mechanical stimuli and secretory outcomes. Chen et al. (2025) identified tension gradients from the cell periphery toward the center, suggesting that intracellular mechanics are not uniform [5]. Whether analogous gradients exist for active GTPases, calcium, or lipid signaling, and how these spatial patterns integrate to regulate localized trafficking decisions, represents an exciting area for future work. This spatial heterogeneity raises the possibility that cells exploit positional information to direct trafficking events to specific subcellular regions based on local mechanical conditions.

Mechanical regulation should not be viewed as an independent layer operating in parallel to biochemical control of secretion, but as one that converges with canonical signaling pathways at specific molecular nodes. Temporal integration is one such challenge: YAP/TAZ‐dependent transcription regulates secretory and ECM protein expression over hours to days, whereas post‐translational mechanisms such as GTPase activation or phosphorylation act within minutes, and how these layers are coordinated remains unclear. Cells also integrate mechanical stress with growth factor, nutrient, and hypoxia signaling, for instance via mTOR, though the nature of this interplay (synergistic, antagonistic, or parallel) is poorly defined. AMPK exemplifies this convergence directly: beyond its role in mitotic Golgi disassembly, AMPK is the kinase through which the Src–FAK–AMPK–GBF1 axis couples mechanical cues to cellular energy status, as reflected by the fact that pharmacological AMPK activation (glucose starvation, metformin) recapitulates the effect of soft substrates on Golgi sorting. A comparable convergence occurs in EV biogenesis, where mechanical inputs are relayed through Akt, ROCK1, and YAP, regulators with well‐established biochemical roles beyond mechanotransduction. A major challenge for future studies will be to determine how mechanical and biochemical inputs are integrated at these and other shared nodes, and how their relative contribution varies across secretory steps and cell types.

The existence of feedback mechanisms linking secretion and matrix mechanics presents both a conceptual challenge and a therapeutic opportunity. Positive feedback between ECM stiffening and enhanced secretion underlies fibrosis and cancer progression, yet it remains unknown whether cells possess intrinsic negative feedback mechanisms to prevent runaway stiffening, and if so, whether these mechanisms could be therapeutically reinforced. Understanding the design principles underlying these feedback circuits could reveal why certain tissues are more susceptible to pathological stiffening than others.

Most insights into mechanosensitive secretion derive from cultured cell systems, leaving open critical questions about physiological relevance in vivo. Determining how these mechanisms operate within the architectural and mechanical complexity of tissues will require innovative experimental approaches. Tissue‐specific knockout models of components of mechanotransduction pathways that regulate trafficking, such as conditional deletions of STIM1, Rac1, or PKD in defined cell types, could provide critical insights into their physiological and pathological roles. Additionally, different cell types may exhibit distinct mechanosensitive secretory profiles. Fibroblasts, epithelial cells, endothelial cells, and immune cells all encounter unique mechanical contexts and may have evolved specialized responses. Systematic comparative studies could help distinguish universal mechanosensitive principles from cell type‐specific adaptations. The impact of aging also deserves attention: tissue stiffening and altered ECM composition are characteristic of aging [74]. Whether these changes modify cellular mechanosensitivity and contribute to age‐associated pathologies remains an open and potentially transformative question.

Progress in addressing these questions will depend critically on new technological capabilities. At present, the mechanobiology of organelles is studied almost exclusively in cultured cells, and methods for measuring organelle mechanics in living animals are lacking. Combining intravital imaging with advanced biosensors could enable direct visualization of organelle deformation and tension in vivo. Likewise, the development of organelle‐specific mechanical actuators could allow the selective application of force to intracellular structures. Although optogenetic systems such as LIMER have proven effective in cultured cells [44], translating these approaches to living organisms would represent a major leap forward. Machine learning also holds considerable promise for this field. By integrating high‐content imaging of trafficking dynamics with computational modeling, artificial intelligence could identify subtle mechanosensitive phenotypes and trafficking signatures that elude human detection. Such approaches could accelerate the discovery of novel regulators and mechanisms.

From a therapeutic standpoint, translating mechanotherapy into clinical practice presents formidable challenges that must be addressed for these insights to benefit patients. Although the concept of normalizing tissue mechanics is compelling, effective and non‐invasive delivery remains a hurdle. Small molecules targeting ECM crosslinking enzymes, as well as physical therapy or device‐based mechanical stimulation, represent potential strategies but require further optimization. Defining appropriate therapeutic windows will be equally critical, as early intervention to prevent pathological stiffening may be more effective than attempting to reverse established fibrosis or tumor desmoplasia. Given the pleiotropic nature of mechanotransduction pathways, specificity represents a key concern. Broad inhibition of central nodes such as FAK or ROCK could produce undesirable side effects. The development of context‐specific inhibitors, for example compounds that selectively block mechanosensitive FAK activation without affecting growth factor‐induced signaling, could improve therapeutic precision and safety.

The convergence of molecular discoveries, technological innovations, and clinical imperatives positions mechanosensitive secretion as a field poised for transformative advances. Addressing these outstanding questions will require interdisciplinary collaboration spanning cell biology, biophysics, materials science, and clinical medicine. As our understanding deepens, integrating mechanical and molecular perspectives promises not only to illuminate fundamental principles of cellular organization but also to unlock new therapeutic strategies for some of the most challenging diseases of our time.

## 6 Conclusions

Over the past decade, our conception of the secretory pathway has been transformed. Once regarded as an autonomous biochemical assembly line, it is now clear that intracellular trafficking is intimately attuned to the physical forces and mechanical properties of the cellular microenvironment. From the initiation of export sites at the ER to Golgi remodeling, cargo sorting, plasma membrane delivery, and even exosome release, every step of the secretory process is finely tuned by mechanical cues [9, 19, 32, 38, 41, 52, 64]. The recognition that organelles such as the Golgi apparatus function as mechanically regulated hubs, encompassing both directly mechanosensitive elements, such as DAG‐sensor recruitment under applied force, and components that respond indirectly to substrate‐derived mechanical signals, has expanded the scope of mechanotransduction beyond the cell periphery, to encompass the entire endomembrane system [52, 57].

Together, the studies discussed above converge on a common framework for mechano‐secretory regulation, organized around three interconnected stages. First, mechanical cues from the ECM and substrate stiffness are sensed at the plasma membrane and transmitted intracellularly through integrins, focal adhesions, and membrane tension, as well as through direct mechanosensitive channels on organelle membranes. Second, these signals are locally decoded by the secretory organelles themselves, particularly the Golgi apparatus and ER exit sites, which act as mechanically regulated checkpoints, predominantly through indirect mechanotransduction, with direct organelle‐level mechanosensing documented in select cases, that determine cargo sorting, retention, or degradation. Third, the resulting changes in secretory output feed back onto the ECM and tissue mechanics, closing the mechano‐secretory loop introduced above and linking organelle‐level regulation to tissue‐scale physiology and disease. This framework reframes the secretory pathway not as a fixed assembly line but as a tunable, mechanically responsive system. An important distinction emerging from these studies is that mechanical regulation of secretion can occur at several separable levels. Mechanical inputs may increase the capacity for cargo export, as reflected by ERES or CARTS formation; alter cargo routing, as shown by the stiffness‐dependent switch between secretion and lysosomal degradation; determine the spatial destination of secretion through adhesion‐associated exocytic hotspots; or change the composition and functional properties of EVs. These processes should therefore not be treated as interchangeable measures of a single “secretory response.” Indeed, a mechanical stimulus could increase carrier formation without necessarily changing the fate of all cargoes, or alter cargo composition without substantially affecting vesicle abundance. This distinction may help reconcile apparently divergent findings across studies and suggests that mechanical regulation is better viewed as control over multiple dimensions of secretory output rather than as a binary increase or decrease in secretion.

This mechanosensitivity depends on a sophisticated network of molecular mechanisms that translate external forces into intracellular responses. Integrins and focal adhesions act as primary mechanoreceptors, converting mechanical stress into biochemical signals through the activation of small GTPases such as RhoA, Rac1, Rab6, Rab8, and ARF1. These, in turn, trigger downstream kinase cascades involving FAK, Src, Akt, PKD, and AMPK, while simultaneously reshaping the cytoskeleton through actin remodeling and microtubule acetylation [9, 11, 19, 29, 38, 55, 61, 64]. The reciprocal crosstalk between mechanotransduction and metabolism further enriches this regulatory landscape: mechanical forces not only tune metabolic pathways, including glycolysis, lipid synthesis, and mitochondrial dynamics, but also cellular metabolism, in turn, regulates cytoskeletal dynamics, adhesion structures, and ECM remodeling, creating bidirectional feedback loops that coordinate tissue mechanics with energetic demands [52]. Lipid signaling intermediates, including DAG and lipin‐1, participate in adjusting membrane composition, and curvature, whereas transcriptional regulators like YAP/TAZ and SREBP ensure long‐term adaptation of the secretory system [11, 18, 19, 52, 75]. Collectively, these pathways converge on the trafficking machinery to control vesicle biogenesis, organelle positioning, membrane tension, and cargo fate.

The functional implications of this integration are profound. Mechanosensitive secretion enables cells to dynamically adjust their output according to environmental demands, reinforcing the ECM when tension increases, modulating adhesion strength under stress, redirecting cargo toward lysosomal degradation when attachment is lost, or releasing exosomes carrying context‐dependent signals. This adaptability supports diverse physiological processes, from tissue development and homeostasis to wound healing and immune regulation.

When dysregulated, however, mechanosensitive secretion contributes to disease. Cancer cells exploit stiff microenvironments to enhance secretion of pro‐tumorigenic factors and exosomes, thereby sustaining growth and metastasis [9, 24, 27, 64, 76, 77]. In fibrosis, pathological tissue stiffening creates a self‐perpetuating loop in which increased rigidity stimulates excessive ECM deposition [27]. Cardiovascular disorders, characterized by altered mechanical loads, and neurodegenerative diseases, where subtle shifts in tissue mechanics perturb organelle homeostasis, may plausibly involve aberrant mechanical signaling within the secretory apparatus [25, 78]. Understanding how these maladaptive processes arise offers new opportunities for therapeutic intervention.

The field now stands at a pivotal intersection where organelle biology, mechanotransduction, and cell‐ECM interactions converge. Emerging technologies are driving this transformation. Optogenetic actuators capable of applying localized forces to organelles, super‐resolution imaging of trafficking dynamics, and tunable hydrogel systems that recapitulate physiological stiffness are revealing mechanosensitive phenomena with clarity. In parallel, omics approaches and high‐throughput genetic screens are mapping the global networks of mechanosensitive regulators that govern secretion.

Future progress will depend on extending these discoveries beyond in vitro models to more physiologically relevant systems. In vivo validation, through tissue‐specific manipulations and advanced imaging, will be essential to understand how mechanical forces regulate secretion in complex tissues. Dissecting cell type‐specific mechanisms and integrating mechanosensitive secretion with other signaling axes such as metabolism, growth factor signaling, and immune regulation represent critical next steps. Translationally, these insights could pave the way for new therapeutic paradigms: restoring normal tissue mechanics (“mechanical normalization”), selectively inhibiting mechanotransduction nodes, or fine‐tuning trafficking machinery to rebalance secretory output in disease. The most effective strategies may prove to be those acting on both fronts simultaneously, reshaping the mechanical properties of the tissue microenvironment while also targeting the intracellular machinery responsible for sensing and transducing these physical cues. Such a dual approach could break the pathological feedback loop between a dysregulated ECM and a hypersensitized cellular mechanosensory response, offering synergistic benefits beyond what either intervention could achieve in isolation.

Ultimately, acknowledging that cargo is under tension reframes one of cell biology's oldest paradigms. The secretory pathway should no longer be viewed as a passive conduit for material transport, but as an active sensor and integrator of mechanical information. By dynamically adjusting cellular logistics to the physical context of the tissue, it ensures homeostasis under stress yet, when perturbed, drives pathology. Incorporating this mechanical dimension into our understanding of secretion not only enriches fundamental cell biology but also opens new horizons for precision medicine, where manipulating mechanics may one day be as powerful as targeting genes or enzymes.

## Author Contributions

D.R. and R.R. conceived, conceptualized, and wrote the manuscript. A.C., A.F.D., and M.L.M. reviewed the manuscript, curated the references, and prepared the figures. S.P. reviewed the work and provided conceptual insights.

## Conflicts of Interest

The authors declare no conflicts of interest.

## Data Availability

The data that support the findings of this study are available from the corresponding author upon reasonable request.
